# Can Enhanced Nutrition Knowledge Improve Adherence to the Mediterranean Diet Among Physically Active Individuals? Evidence From a Cross‐Sectional Study

**DOI:** 10.1002/fsn3.70327

**Published:** 2025-07-08

**Authors:** Hatice Kübra Barcın Güzeldere, Oğuzhan Aydın, Ahmet Bedirhan Kumbasar, Nagihan Sinan, Zeynep Zülal Yıldız, Bashir A. Lwaleed

**Affiliations:** ^1^ Faculty of Health Science, Department of Nutrition and Dietetic Istanbul Medeniyet University Istanbul Türkiye; ^2^ School of Health Science University of Southampton Southampton UK

**Keywords:** MEDAS, Mediterranean diet, NSKQ, nutrition knowledge, physically active individuals

## Abstract

The Mediterranean diet (MD) health benefits are well‐known for the general population and athletes alike. Nutrition knowledge can affect food choices and dietary habits. This study aimed to evaluate the relationship between sports nutrition knowledge and adherence to the MD in physically active individuals (PAI). A total of 400 PAI participated in our cross‐sectional study, 74.5% women and 25.5% men. The survey was performed in person. Information on demographic variables, Nutrition for Sports Knowledge Questionnaire (NSKQ), and Mediterranean Diet Adherence Screener (MEDAS) was collected. Among PAIs, there was low nutritional knowledge (67.8%) and low adherence to the MD (65.5%). There was a significant difference between the levels of sports nutrition knowledge of PAI and MD adherence (Low NK‐MEDAS = 6.05 ± 2.50, Very good NK‐MEDAS = 7.28 ± 2.06, *F* = 7.252, *p* < 0.01). In addition, the relationship between subdimension scores and adherence to the MD level were; total NSKQ score (*r* = 0.245, *p* < 0.01), macronutrients (*r* = 0.22, *p* < 0.01), micronutrients (*r* = 0.156, *p* < 0.01), sports nutrition (*r* = 0.202, *p* < 0.01), supplements (*r* = 0.217, *p* < 0.01), and alcohol (*r* = 0.182, *p* < 0.01), respectively. In conclusion, nutritional knowledge should increase adherence to the MD among PAI. Thus, nutrition education and personalized nutrition counseling could help to improve nutritional knowledge and PAI's adherence to the MD.

## Graphical abstract

Physically active individuals (PAI) have low nutritional knowledge. PAI also showed low adherence to the Mediterranean diet. Nutritional knowledge is related to adherence to Mediterranean diet among PAI.

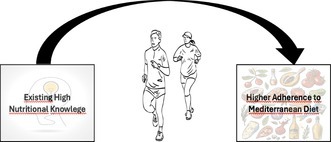

## 1 Introduction

Nutrition is vital for disease prevention, lifelong health, and athletic performance. Adequate and balanced nutrition can enhance athletic performance, aid recovery, prevent injuries, and maintain health among athletes (Thomas et al. 2016). However, many athletes do not adhere to a healthy diet and fail to meet adequate nutrient intake levels (Moss et al. 2023). Healthy dietary habits are closely linked to the consumption of nutritious homemade meals. Moreover, higher nutritional knowledge has a significant impact on maintaining a healthy diet (Jessri et al. 2010). Nutrition knowledge influences food choices and healthy dietary habits (Alaunyte et al. 2015; Davar 2012). Furthermore, eating behavior can be driven by nutrition knowledge, which also affects athletic performance (Trakman et al. 2018). Higher nutritional knowledge is often associated with factors such as female gender, higher athletic caliber, and involvement in body‐building sports (Raymond‐Barker et al. 2007). To optimize athletic performance, not only are nutritional factors crucial, but also are sports‐nutrition‐specific strategies related to training and competition (Sharman 2009). While macronutrients such as carbohydrates, protein, and lipids play a significant role in body metabolism regulation, energy utilization, and overall performance, micronutrients such as iron, vitamins, and minerals also significantly impact athletic achievement (Lukaski 2001). Nutrition knowledge has been shown to promote healthy food choices and the development of healthy dietary habits among adults and specific groups (Akkartal and Gezer 2020; Noronha et al. 2020).

A growing body of research on the Mediterranean Diet (MD) highlights it as the most prudent dietary pattern, offering numerous health benefits to the general population and athletes. The MD is characterized by a high intake of vegetables, pulses, fresh fruits, unrefined cereals, nuts, and especially olive oil, along with a moderate intake of fish and dairy products, a low intake of red meat, and moderate consumption of alcohol, mainly red wine, at main meals. Seasonal and fresh vegetables and fruits are essential components of the MD (Trichopoulou and Lagiou 1997). The plant‐based nature and variety of foods in the MD are thought to contribute to its health benefits. These include vegetables and fruits rich in vitamins B, C, A, K, and E, and minerals such as selenium, calcium, magnesium, and potassium. Fish, a fundamental component of the MD, is beneficial due to its high omega‐3 content. Olive oil, used in all meals including cooking, contains omega‐9 and squalene, which diversify the diet's nutrient profile. Furthermore, the occasional consumption of red wine contributes to the intake of resveratrol, supporting the antioxidant system (Gantenbein and Kanaka‐Gantenbein 2021; Nani et al. 2021; Singh et al. 2022).

The MD ingredients collectively help maintain the body's antioxidant balance. While adequate antioxidant intake is crucial for metabolic pathways, maintaining a balance between antioxidants and reactive oxygen species (ROS) is also essential for regulating metabolic processes and athletic performance. During training or competition, increased energy metabolism and oxygen requirements lead to higher ROS production (Clarkson 1995). To counteract the detrimental effects of ROS, consuming adequate, balanced, and healthy foods rich in antioxidants is particularly important for athletes (Braakhuis and Hopkins 2015; de Sousa et al. 2017; Galli and Visioli 2001). Components of the MD, such as omega‐3, vitamin C, resveratrol, and selenium, help maintain antioxidant balance in athletes (Clemente‐Suárez et al. 2023). Besides the general health benefits, the MD could also improve sports performance (Baker et al. 2019; Calella, Gallè, Di Onofrio, et al. 2022). Additionally, raising awareness of nutrition knowledge may enhance adherence to the Mediterranean diet among athletes (Philippou et al. 2017). The literature has demonstrated a relationship between nutritional knowledge and MD adherence across different populations (Aureli and Rossi 2022; Bonaccio et al. 2013; Neshatbini Tehrani et al. 2021). Higher levels of nutritional knowledge correlate with better dietary habits (Philippou et al. 2017; Spronk et al. 2015). Costantino et al. (2023) found that higher nutritional knowledge was highly linked to adhering to the MD‐like diet, including consuming more vegetables and fruits. Moreover, Kontele et al. (2021) showed that a high adherence to the MD was correlated with a healthier BMI among adolescent female gymnasts. It was also found that higher adherence to the MD was positively related to higher nutrition knowledge and had an inverse relationship with BMI scores (Elmskini et al. 2024). A study highlighted that athletes with higher nutritional literacy are more likely to adopt the MD patterns. This was found to enhance athletic performance and general health status (Morelli et al. 2021). Melguizo‐Ibáñez et al. (2023) further elucidated that adherence to the MD improves mental and psychological well‐being and reduces external pressures related to physical appearance, particularly among less active participants. Higher nutritional knowledge drives choices for healthy food (Taylor et al. 2019); it contributes to increased athletes' adherence to the MD. To fully realize the benefits of the MD, higher nutritional knowledge is crucial among athletes. The present study aims to examine the relationship between sports nutrition knowledge and adherence to the MD among athletes, thus addressing a significant gap in the current literature about an important health‐related issue.

## 2 Methods

### 2.1 Study Setting

This cross‐sectional, multi‐center study was conducted between January 2023 and April 2023 in Türkiye. Participants from thirteen different sports centers/sports clubs were invited to take part in the study. These were Kağıtspor, Eğitim Spor, Ürgüpspor, Dürümle TED Ankara Kolejliler, Tarım Kredi Gençlik, Cadence, Yalçın Kızılay Sports Club, Mac Fit, KYK Mimar Sinan Sports Club, İBB fitness saloon, Athletic Fitness Sports Club, and Rıdvan Dilmen Sports Facility. The sports centers were determined based on their locations and popularity. Informed consent was obtained from all participants. The researchers conducted the face‐to‐face survey.

### 2.2 Participants

A total of 400 physically active individuals aged between 18 and 65 years participated in the study. Inclusion criteria included being actively involved in a sport, regularly attending sports centers or sports groups, and having either a professional or non‐professional sports career. Individuals who benefit from the facilities offered at the gym, such as weightlifting, fitness, step‐aerobics, or a group sport player, such as basketball or volleyball, were invited to the study. Ethical approval was obtained from the Istanbul Medeniyet University Göztepe Training and Research Hospital Clinical Research Ethics Committee (Reference No: 2022/0761). Based on the national data reported by the Turkish Statistical Institute (TUIK 2022), the prevalence of individuals engaging in regular physical activity in Türkiye was 18.4% in 2022. Assuming a 95% confidence level and a 5% margin of error, the minimum required sample size for ensuring the statistical significance of the study was calculated to be 231 individuals. The study was conducted on a total of 400 participants in case of dropout. A post hoc power analysis revealed that, with this sample size, the study achieved a statistical power of approximately 99.99%, indicating a very high probability of detecting a true effect if one exists.

### 2.3 Survey

The survey consisted of 89 questions divided into three sections. The first section addressed sociodemographic variables (such as age, gender, education, marital status, and economic status below minimum wage/minimum wage/upper minimum wage). The second section included the Nutrition for Sports Knowledge Questionnaire (NSKQ), and the third section assessed adherence to the Mediterranean diet using the Mediterranean Diet Adherence Screener (MEDAS). The survey was designed by the researchers and completed by the participants in person.

### 2.4 Nutrition for Sports Knowledge Questionnaire (NSKQ)

The NSKQ, developed by Gina Louise Trakman et al. (Trakman et al. 2018) was used to evaluate nutritional knowledge among adult athletes. The Turkish version, validated and verified for reliability by Çırak and Çakıroğlu (2019), contains 68 items across six sub‐dimensions:

1. Weight control (3 items)
2. Macronutrients (22 items)
3. Micronutrients (12 items)
4. Sports nutrition (11 items)
5. Supplements (11 items)
6. Alcohol (9 items)

Participants responded using a 3‐point Likert scale (agree‐disagree‐not sure or effective‐not effective‐not sure). The total score was calculated based on correct responses, with 68 items accounting for 100 points, each correct answer worth 1.47 points. Scores were categorized as follows: 0%–49% (poor nutritional knowledge), 50%–56% (moderate nutritional knowledge), 66%–75% (good nutritional knowledge), and 76%–100% (very good nutritional knowledge).

### 2.5 Adherence to Mediterranean Diet (MEDAS)

The MEDAS scale, developed by Papadaki et al. (2018), was validated in Turkish by Özkan Pehlivanoğlu et al. (2020). It consists of 14 questions assessing the frequency of food consumption (12 items) and food consumption habits (2 items). The total score is calculated by awarding 1 point for each correct answer and 0 points for incorrect answers, resulting in a total score range of 0–14. A score of 7 or above indicates acceptable adherence to the MD, while a score of 9 or above suggests high adherence.

### 2.6 Statistical Analysis

Data analysis was performed using SPSS 26. Normality was assessed using the Kolmogorov–Smirnov and Shapiro–Wilk tests, skewness–kurtosis values, and histograms. Descriptive variables were presented as mean ± standard deviation (SD) and percentage (%). The participants' scale results were compared using One‐Way ANOVA for normally distributed data among three different groups. A bivariate correlation method was used to determine the relationship between nutrition knowledge and MEDAS scores.

## 3 Results

Participants' demographic variables are shown in Table 1. The mean age of the PAI was 23.60 ± 5.11 years. Most participants had graduated from university and reported a high economic status. Additionally, 36.8% had been involved in sports for more than five years.

**TABLE 1:** Demographic variables of the athletes.

	PAI (*n* = 400)
Age (mean ± SD)	23.60 ± 5.11
Gender (%, *n*)	
Men	25.5, 102
Women	74.5, 298
Education status (%, *n*)	
Illiterate	1, 4
Primary school	0.2, 1
High school	23.5, 94
Graduate	75.3, 301
Economic status (%, *n*)	
Low	35, 140
Moderate	15.8, 63
High	49.2, 197
How long have you been interested in sports? (%, *n*)	
0–6 months	18.5, 74
6–12 months	12.8, 51
1–2 years	19.5, 78
2–5 years	12.5, 50
> 5 years	36.8, 147

Abbreviation: PAI, physically active individuals.

Table 2 shows the participants' scale results. According to the NSKQ, 67.8% of participants had a low level of nutrition for sports knowledge, with all subdimensions showing low levels of knowledge. Moreover, 65.6% of participants exhibited low adherence to the MD, as indicated by the mean MEDAS score.

**TABLE 2:** The Nutrition for Sport Knowledge Questionnaire and MEDAS scores of participants.

	PAI (*n* = 400)
	**Mean ± SD**
The Nutrition for Sport Knowledge Questionnaire (NSKQ)	41.66 ± 15.43
Weight management	1.57 ± 1.40
Macronutrients	15.07 ± 6.87
Micronutrients	8.15 ± 3.78
Sports nutrition	5.78 ± 3.16
Supplements	4.92 ± 2.95
Alcohol	5.92 ± 3.70
Classification of NSKQ	**% (*n*)**
Low	67.8 (271)
Moderate	27 (108)
Good	4 (16)
Very good	1.2 (5)
	**Mean ± SD**
MEDAS	6.47 ± 2.21
	**% (*n*)**
Low Mediterranean Diet Adherence	65.5 (262)
Moderate Mediterranean Diet Adherence	25.3 (101)
High Mediterranean Diet Adherence	9.2 (37)

Abbreviations: MEDAS, Mediterranean Diet Adherence; NSKQ, Nutrition of Sports Knowledge Questionnaire; PAI, physically active individuals.

The relationship between nutritional knowledge and adherence to the MD is shown in Table 3. Participants with low or moderate nutritional knowledge demonstrated low adherence to the MD. In contrast, those with good or very good nutritional knowledge had moderate adherence to the MD (*p* < 0.01). A weak positive correlation was observed between sports nutrition knowledge and adherence to the MD (*r* = 0.245, *p* < 0.01). It was also noted that participants with higher adherence to the MD had better scores in the subdimensions of macronutrients, sports nutrition, and alcohol (*p* < 0.01). Additionally, higher scores in the subdimensions of macronutrients, micronutrients, sports nutrition, supplements, and alcohol were associated with greater adherence to the MD (*p* < 0.01).

**TABLE 3:** The relationship between nutrition knowledge and adherence to the Mediterranean diet.

	MEDAS‐total	*p*
	Mean ± SD	
NSKQ‐total		***F* = 7.252** ***P* < 0.01**
Low	6.05 ± 2.50^a^	
Moderate	6.01 ± 2.09^a^	
Good	6.49 ± 1.75^a^	
Very good	7.28 ± 2.06^a^	

	MEDAS classification			*p*	*r*
	Low	Moderate	High		
	% (*n*)				
NSKQ‐total				***χ*2 = 25.43**. ***p* =0.002**	***r* = 0.245** ***p* < 0.01**
Low	72.7 (197)	20.7 (56)	6.6 (18)		
Moderate	52.8 (57)	31.5 (34)	15.7 (17)		
Good	43.8 (7)	50 (8)	6.3 (1)		
Very good	20 (1)	60 (3)	20 (1)		
NSKQ‐weight management	1.05 ± 0.72	0.86 ± 1.46	1.30 ± 1.46	*F* = 1.816 *p* = 0.164	*r* = 0.067 *p* = 0.179
NSKQ‐macronutrients	10.50 ± 6.15^a^	9.07 ± 6.01	12.11 ± 5.82^a^	***F* = 7.550** ***p* = 0.01**	***r* = 0.222** ***p* =0.001**
NSKQ‐micronutrients	3.99 ± 3.47	4.66 ± 4.15	7.00 ± 3.27	*F* = 1.575 *p* = 0.208	***r* = 0.156** ***p* = 0.002**
NSKQ‐sports nutrition	2.94 ± 2.25^a^	3.43 ± 2.01^a^	4.32 ± 2.57	***F* = 9.137** ***p* < 0.01**	***r* = 0.202** ***p* < 0.01**
NSKQ‐supplements	2.52 ± 2.78^a^	4.17 ± 2.65^a^	3.29 ± 2.47	***F* = 6.731** ***p* < 0.01**	***r* = 0.217** ***p* < 0.001**
NSKQ‐alcohol	3.36 ± 3.26^a^	3.80 ± 4.04^a^	4.67 ± 3.09	***F* = 5.071** ***p* = 0.007**	***r* = 0.182** ***p* < 0.001**

*Note:* *p* < 0.05 and *p* < 0.01 statistically significant. bold values show significance at level *p* < 0.05.

Abbreviations: *F*, One‐way‐ANOVA test; MEDAS, Mediterranean Diet adherence; NSKQ, Nutrition of Sports Knowledge Questionnaire; *χ*2, Chi‐square test.

^a^ Post hoc analysis of groups Tukey, *p* < 0.05.

## 4 Discussion

This multi‐center cross‐sectional study aimed to explore the relationship between sports nutrition knowledge and adherence to the Mediterranean diet (MD) among PAI. It was found that a significant proportion of the PAI, specifically 67.8%, possessed a low level of nutritional knowledge and similarly exhibited low adherence to the MD. Furthermore, there was a weak positive correlation between sports nutrition knowledge and MD adherence, suggesting that PAI with higher levels of nutritional knowledge were more likely to adhere to the MD. Additionally, the subdimension scores of the Nutrition for Sports Knowledge Questionnaire (NSKQ) also showed a weak positive relationship with adherence to the Mediterranean diet.

Optimal nutrition is essential for achieving peak sports performance. Nutritional knowledge can play a pivotal role in enhancing dietary diversity and ensuring adequate nutrient intake by influencing dietary choices. Various studies have highlighted that athletes often have inadequate diets alongside insufficient nutritional knowledge (Heikura et al. 2017; Heydenreich et al. 2017; Masson and Lamarche 2016; Spendlove et al. 2012). Bird and Rushton (Bird and Rushton 2020) found that young athletes particularly lacked knowledge about supplements and recommended dietary intakes. Werner et al. (2022) demonstrated that athletes tend to have limited nutrition knowledge, leading to poor dietary decisions. A systematic review by (Janiczak et al. 2022) reported a weak‐to‐moderate positive relationship between nutrition knowledge and dietary choices. Another study concluded that athletes generally possess low nutritional knowledge and should be educated on avoiding unreliable information sources and unprescribed supplements (Vázquez‐Espino et al. 2022). It was shown in the literature, most athletes have low nutritional knowledge. The literature consistently indicates that most athletes lack adequate nutritional knowledge. Factors such as unreliable information sources, educational levels, and economic status may influence this knowledge. In this study, despite the athletes having good educational backgrounds and considering their economic status moderate‐to‐high, their nutritional knowledge levels were low. This suggests that the sources of their nutrition information might be a contributing factor, although this aspect was not specifically investigated.

Adequate nutrient intake is crucial for achieving optimal sports performance. An athlete's diet should provide sufficient energy, carbohydrates, proteins, fats, vitamins, and minerals to support muscle building, prevent injuries, replenish energy, and reduce fatigue. In recent years, the MD has been recognized for its health benefits, diverse food options, and rich nutrient content. Calella et al. (Calella, Gallè, Di Onofrio, et al. 2022) found that athletes have similar adherence to the MD as the general population, suggesting that the MD can be adapted to meet athletes' nutritional needs (Bifulco et al. 2019). The MD also offers adequate antioxidants, which are essential in countering oxidative stress. Watson et al. (2005) compared the effects of naturally high and low antioxidant intake on exercise response, concluding that athletes' antioxidant needs may increase with high‐intensity exercise. They noted that a diet rich in antioxidants could meet these needs and protect against exercise‐induced oxidative stress. The findings on athletes' adherence to the MD are mixed (Calella, Gallè, Cerullo, et al. 2022; Muros and Zabala 2018). Although there are insufficient studies to definitively recommend the MD for enhanced performance, Baker et al. (2019) demonstrated that a short‐term MD could improve endurance exercise performance in as little as four days. Similarly, Ficarra et al. (2022) found that adherence to the MD could aid in developing specific strength, endurance, and anaerobic capacity without altering body composition. Another study indicated that the MD increased aerobic capacity in adolescent athletes (Helvacı et al. 2023). The MD's wide range of foods, including carbohydrates for energy, proteins for muscle protection and rebuilding, a low inflammatory index to combat oxidative stress, vitamins and minerals from vegetables and fruits, and healthy fats from olive oil and fish, make it an accessible dietary pattern for athletes. These characteristics suggest that the MD can enhance athletic performance (Kaufman et al. 2023).

In this study, participants showed low adherence to the MD. Although factors such as the participants' relatively young age, educational background, and economic status may influence adherence levels, the low nutritional knowledge observed among participants likely contributes to these results. Continuous nutrition education programs and personalized nutritional counseling could help improve adherence to the MD among athletes. Furthermore, incorporating the benefits of the MD for athletes' health and performance into guidelines could enhance its adoption.

It is well‐established that food choice and diet quality are closely linked to nutritional knowledge (Janiczak et al. 2022; Montecalbo and Cardenas 2015; Spronk et al. 2015). Consequently, the level of nutritional knowledge can significantly impact adherence to the MD among athletes. Werner et al. (2022) found that low nutritional knowledge often results in inappropriate food choices among athletes, raising concerns about the potential negative effects on athletic performance. Similarly, another study indicated that many athletes possess low levels of nutritional knowledge, which may influence their food preferences (Heikkilä et al. 2017). Jagim et al. (2021) examined perceptions of dietary recommendations among collegiate athletes and discovered that these athletes generally had low nutritional knowledge and misconceptions about dietary requirements. Conversely, a high level of nutritional knowledge has been shown to positively affect adherence to the MD. Aureli and Rossi (2022) found that individuals with the lowest levels of nutritional knowledge also had the lowest adherence to the MD, while those with the highest levels of nutritional knowledge had the greatest adherence to the diet. According to existing literature, there is a notable relationship between nutritional knowledge and adherence to the MD, suggesting that nutritional knowledge correlates with healthy dietary habits in the general population.

Our findings are consistent with previous research. The results of our study indicate that PAIs’ nutritional knowledge is associated with their adherence to the MD. Participants with higher nutritional knowledge scores demonstrated a greater level of adherence to the MD. The literature also suggests that adherence to the MD can be enhanced through nutrition education interventions (Philippou et al. 2017). Nascimento et al. (2016) indicated that nutritional counseling is an effective strategy for improving body composition, eating behavior, and nutritional knowledge among athletes. Ongoing nutrition education sessions and personalized nutritional counseling could therefore support better adherence to the MD among athletes.

While the present study has several strengths, we acknowledge that there are a few limitations. A notable strength is the evaluation of the relationship between nutrition knowledge and MD adherence among 400 PAI using valid and reliable questionnaires. However, there are also limitations. One such limitation is the lack of data on 3‐day food consumption and food frequency, which would have allowed for a more detailed assessment of the athletes' nutritional intake and adherence to the MD. Furthermore, we did not inquire about the sources of the athletes' nutritional information. Additionally, we were unable to analyze the participants' body composition. One key limitation of the present study was the reliance on self‐reported data regarding nutrition knowledge and adherence to the MD. This may introduce reporting bias and affect the accuracy of the findings. In addition, the lack of information on participants' weight limited the ability to account for its potential influence on dietary behavior, particularly among individuals who may be actively attempting weight loss. This concern is especially relevant considering that over 30% of the study population had not engaged in any form of physical activity for more than 12 months, a factor that could have further impacted both nutritional habits and self‐perception. Future studies should explore the potential interactions between body composition, food consumption, nutritional knowledge, and adherence to the MD among athletes.

In conclusion, maintaining a successful sports career requires adequate, balanced, and healthy nutrition. Our study found that PAI generally possess low levels of nutritional knowledge and low adherence to the MD. We also found that nutrition knowledge had a weak positive relationship with MD. Moreover, participants with higher adherence to the MD diet had better scores in the subdimensions of macronutrients, sports nutrition, and alcohol. Additionally, higher scores in the subdimensions of macronutrients, micronutrients, sports nutrition, supplements, and alcohol were associated with greater adherence to the MD. To foster sustainable and beneficial dietary habits among physically active individuals, efforts should be made to enhance their nutritional knowledge and adherence to the MD. Various nutritional interventions, such as educational sessions, seminars, camps, and interactive activities, could be implemented to improve PAI's levels of nutritional knowledge.

## Author Contributions

**Hatice Kübra Barcın Güzeldere:** conceptualization (lead), data curation (equal), formal analysis (lead), investigation (lead), methodology (lead), project administration (equal), supervision (lead), writing – original draft (lead), writing – review and editing (lead). **Oğuzhan Aydın:** conceptualization (equal), data curation (equal), funding acquisition (equal), investigation (equal), methodology (equal), project administration (equal), writing – review and editing (equal). **Ahmet Bedirhan Kumbasar:** conceptualization (equal), data curation (equal), formal analysis (equal), investigation (equal), methodology (equal), project administration (equal), writing – review and editing (equal). **Nagihan Sinan:** conceptualization (equal), data curation (equal), formal analysis (equal), investigation (equal), methodology (equal), project administration (equal), writing – review and editing (equal). **Zeynep Zülal Yıldız:** conceptualization (equal), data curation (equal), formal analysis (equal), investigation (equal), methodology (equal), project administration (equal), writing – review and editing (equal). **Bashir A. Lwaleed:** supervision (equal), writing – original draft (equal), writing – review and editing (equal).

## Ethics Statement

Ethical approval was obtained from the Istanbul Medeniyet University Göztepe Training and Research Hospital Clinical Research Ethics Committee (Reference No: 2022/0761).

## Consent

Informed consent was obtained from all participants. Data collection took place between January 2023 and April 2023.

## Conflicts of Interest

The authors declare no conflicts of interest.

## Data Availability

The data supporting the findings of this study can be obtained from the corresponding author, Dr. Hatice Kübra Barcın Güzeldere.
